# Prevalence of Depression and Its Associated Factors among Prisoners in North Wollo Zone, Northeastern Ethiopia: A Cross-Sectional Study

**DOI:** 10.1155/2023/2612900

**Published:** 2023-06-26

**Authors:** Kindie Mekuria Tegegne, Solomon Moges Demeke, Mekonnin Tesfa Lemma, Ayelign Mengesha Kassie

**Affiliations:** ^1^Department of Psychiatry, College of Health Science, Woldia University, P.O. Box 400, Woldia, Ethiopia; ^2^School of Nursing, College of Health Sciences, Woldia University, P.O. Box 400, Woldia, Ethiopia; ^3^School of Public Health, Faculty of Medicine, The University of Queensland, Australia

## Abstract

**Background:**

Depression is a psychiatric disorder that is characterized by persistent sadness and a lack of interest or pleasure in previously rewarding and enjoyable activities. It is one of the leading mental disorders among prisoners worldwide. However, little attention is given to this condition, especially in developing countries. Hence, this study was aimed at assessing the prevalence of depression and its associated factors among prisoners in North Wollo Zone Correctional Institutions, Ethiopia.

**Methods:**

A cross-sectional study was carried out among 407 prisoners from November 20 to December 20, 2020. A simple random sampling technique was employed to select the study participants, and the Patient Health Questionnaire-9 (PHQ-9) was utilized to measure the prevalence of depression among prisoners. Data analyses were done using SPSS version 20 software program. Descriptive and inferential statistics including bivariate and multivariable regression analyses were run to assess the association between depression and the independent variables, and a *p* value of less than 0.05 was taken to declare statistically significant values.

**Results:**

A total of 407 prisoners participated in the study, making the response rate 96.9%. The mean age of the participants was 31.7 ± 12.83. Forty-one percent of them were between the ages of 18 and 27 years. In this study, the prevalence of depression was 55.5%. Age 38-47 (AOR = 4.29; 95%CI = 1.51, 12.20), having children (AOR = 2.75; 95%CI = 1.40, 5.42), sentences for 5-10 years and over 10 years (AOR = 6.26; 95%CI = 3.19, 12.30 and AOR = 7.71; 95%CI = 3.47, 17.17, respectively), having a history of mental illness (AOR = 5.22; 95%CI = 2.39, 11.36), having two or more stressful life events (AOR = 6.61; 95%CI = 2.73, 15.96), and poor social support (AOR = 8.13; 95%CI = 3.43, 19.27) were significantly associated with depression.

**Conclusions:**

In this study, more than half of the study participants were found having depression which is relatively higher compared with other previous studies across the globe. Moreover, different variables including the inmate's age of 38-47 years, having children, a sentence of 5-10 and over 10 years, history of mental illness, having two or more stressful life events, and poor social support were factors significantly associated with depression. Thus, awareness creation for police officers and prison managers about depression screening in prison and treatment programs including psychological counseling and cognitive behavioral therapy for prisoners are recommended.

## 1. Background

Depression is the most important mental health disorder that is characterized by depressed mood, significant weight change, excessive sleep or loss of sleep, a feeling of agitation or psychomotor retardation, fatigue, feelings of guilt, reduced concentration and attention, and attempted suicide [1, 2]. It is a manageable mental illness [3], with an expected prevalence of 4.4% worldwide in 2015 [4]. Nevertheless, the majority of people anguishing from depression are residents of low- and middle-income countries, where there is little concern from both governments and the public about the disease [4, 5]. According to the World Mental Health investigation which was studied in seventeen countries, one in 20 people reported having depression [6]. In addition, the level of severe mental illness was higher among inmates compared to the noninmate population in a study conducted in France [7]; of these conditions, depression was particularly higher in the inmate population, in Ethiopia [8]. These repots are supported by other studies that inmates are five to ten times more prone to depression than the general population worldwide [9, 10], and in low-income countries, the figure is estimated to be even higher [11, 12]. Furthermore, a systematic review conducted in 24 western countries showed that 10.2% of male prisoners and 14.1% of female prisoners experienced depression during their sentence time [13]. In addition, another study conducted in Africa revealed that 23.3% of the inmates were found having depression which is expected to be higher among females and young age groups [14]. Furthermore, in the sub-Saharan African countries, the magnitude of depression among prisoners was found to be 42.2%, and those with lower educational status and unmarried were more affected [12, 15]. Depression results from a complex interaction of social, psychological, and biological factors. In the inmate population, it occurs as a result of overcrowding, different forms of abuse, lack of space to self, isolation from social networks, uncertainty about prospects including their work and relationships, insufficient health services, particularly mental health services in prisons, absence of social support, older ages, and condition of the prison environment [5, 6, 16]. People who have gone through major unfavorable life events are more likely to develop depression, and depression can, in turn, lead to more stress and dysfunction and worsen the affected person's life situation and the depression itself [4, 17]. Depression is among the top morbidity cases in the general population, in Ethiopia [18]. The problem is even higher among prisoners. For instance, a systematic review and meta-analysis studies conducted in the country reported that depression among inmates ranges from 44.45% to 53.40%, and chronic illnesses, history of chat chewing, smoking, alcohol use, and self-killing attempts were also identified as concurrent problems and/or risk factors for this disorder [19, 20]. In addition, Ethiopia's National Mental Health Strategy (2012/13-2015/16) has included prisoners as vulnerable groups alongside persons with severe mental illness, people with substance abuse disorders, children and adolescents, persons living with HIV/AIDS, child-bearing women, victims of violence and abuse, persons with epilepsy, and the elderly who require special consideration when developing mental health services [21]. Therefore, taking evidence-based measures can help keep prisoners healthy and reintegrate them into their prior community when they complete their prison sentence duration [22]. However, despite the presence of some studies which are conducted to assess depression and its associated factors among different groups of the population in Ethiopia, only a few studies are conducted among incarcerated societies when it is compared countrywide. Moreover, there is limited information about the magnitude of depression and its associated factors in the study settings. Hence, this study was aimed at assessing the prevalence of depression and its associated factors among prisoners in North Wollo Zone Correctional Institutions, Northeastern Ethiopia.

## 2. Methods

### 2.1. Study Area and Period

This study was conducted in North Wollo Zone Correctional Institutions from November 20 to December 20, 2021. North Wollo Zone is one of the ten administrative zones of the Amhara region. According to the 2007 Census of Ethiopia, conducted by the Central Statistical Agency (CSA), North Wollo Zone has a total population of 1,500,303, of whom 752,895 are men and 747,408 are women [23]. According to data obtained from the prison administrator's verbal report, the North Wollo Zone has two correctional institutions that are found in Woldia and Lalibela cities. There were a total of 1326 prisoners during the data collection period in the two correctional sites, Lalibela (330) and Woldia (996).

### 2.2. Study Design, Population, and Eligibility Criteria

An institution-based cross-sectional study was employed in this study. The source population includes all prisoners in North Wollo Zone prisons. The study population includes all prisoners who were present during the data collection time in the selected correctional institutions. Prisoners sentenced for being proven guilty were included in this study, whereas prisoners with known mental illness and unable to communicate and critically ill were excluded.

### 2.3. Sample Size and Sampling Procedure

A single population proportion formula was used to calculate the sample size for this study. To get the maximum sample size, we took the prevalence of depression (45.5%) from a previous study [24], with a 5% margin of error and a 95% confidence level, where, nf = [(1.96)^2^ × 0.455 × 0.545)/0.0025] = 381. Thus, by adding a 10% (38) nonresponse rate, the total sample size became 419.21 ≈ 420. Thus, considering a 10% nonresponse, 420 was taken as the final sample size. Then, based on proportional allocation, 105 and 315 study participants from the Lalibela and Woldia prisons, respectively, were included in the study by using a simple random sampling technique. Finally, using the list of prisoners as a sampling frame, study participants were selected by a computer-generated random number.

### 2.4. Study Variables

The outcome variable was depression, and the independent variables were sociodemographic factors (age, sex, religion, marital status, having children, preprison area of residence, ethnicity, educational status, and occupation); health-related variables including mental and bodily health conditions, family history of mental illness, stressful life events, and social support and sociobehavioral and prison environment-related factors including substance use and imprisonment status were included.

### 2.5. Operational Definitions

#### 2.5.1. Depression

Patient Health Questionnaire-9 is a nine-item module with a score ranging from 0 to 27. The scores are divided into five classes where a score of 0–4 shows normal range or “full remission,” 5–9 shows “minimal symptoms,” 10–14 shows major depression with “mild severity,” 15–19 shows major depression with “moderate severity,” and 20 or higher equals major depression with “severe severity”[25]. This was done based on the diagnostic statistical manual of mental disorders' five depression classifications which is guided by the American Psychiatric Association [26]. In addition, PHQ-9 had been translated and validated in Ethiopia previously [27–29]. However, in this study, participants were considered as having depression if they scored five and above for the PHQ-9 questions and not otherwise.

*(1) Chronic Illness*. At least one of these chronic diseases is considered: HIV/AIDS, hypertension, diabetes, cancer, asthma, hypertension, and heart disease [30].

*(2) Social Support*. Social support was measured by the Oslo 3-item social support scale (SSS) [31]. The overall score ranges from 3 to 14 with three large domains or categories: having “poor support” from 3 to 8, “moderate support” from 9 to 11, and “strong support” from 12 to 14 [32].

*(3) Presence of Stressful Life Events*. Individuals had at least one or more life events of stress (associated with a close relative's death, divorce, serious illness or injury in a family member, etc.) in the last four weeks [33].

### 2.6. Data Collection Tool/s

The data were collected using an interviewer-administered questionnaire. The questionnaire includes sociodemographic characteristics, imprisonment status, self-reported mental and physical health problems, substance use status, social support, and stressful life events. Stressful life events were assessed using the life-threatening event [33]. Depression was assessed using the PHQ-9, and the symptoms were evaluated on a scale ranging from zero (not at all) to three (nearly every day) [34].

### 2.7. Data Quality Control

Overall, four BSc psychiatric nurses participated in collecting the data from the prisoners. The data collectors were supervised by one supervisor at each study site. Orientations were given to the data collectors and supervisors on the questionnaire contents and instructions, the objectives of the study, and how to collect data. Pretest was also done on five percent of the sample size (21) of inmates at South Wollo Zone Correctional Institution, Dessie town.

### 2.8. Data Analysis

The data were coded, cleaned, and checked for fullness, entered into EpiData 4.6 version, and exported to Statistical package for Social Sciences version 25 for cleaning and further analysis. Descriptive and inferential statistics were done. Binary and multivariable logistic regression analyses were utilized to identify factors associated with depression. Variables with a *p* value of < 0.25 in binary logistic regression were entered into the multiple logistic regression. The association between the explanatory and outcome variables was determined using adjusted odds ratios at 95% CI. Variables with a *p* value of < 0.05 in the multivariable logistic regression analysis model were considered statistically significant.

## 3. Results

### 3.1. Sociodemographic Characteristics of Respondents

A total of 420 prisoners were interviewed with a response rate of 96.9%. Their ages range from 18 to 99 years with a mean age of 31.7 ± 12.83 SD. Forty-one percent were between the ages of 18 and 27 years. Almost all the respondents were males (96.3%) and Amhara by ethnicity 392 (96.3%). Of the participants, 342 (84%) and 183 (45%) were literate and unemployed, respectively. In addition, about 52.3% of inmates had children (Table 1).

### 3.2. Prevalence of Depression and Psychosocial and Clinical Characteristics of Prisoners

The overall prevalence of depression among the inmate population, as per the Patient Health Questionnaire-9 scored at or above the cut-off value for the depression scale, was 55.5%. In addition, about nine percent had a family history of mental illness (35 (8.6%)), and around one-fifth of them had a history of mental illness (83 (20.4%)). Around three-fourth of study participants had two or more stressful life events (304 (74.7%)). The majority of the prisoners (82.3%) were first-time offenders and the rest had been reincarcerated (Table 2). Furthermore, study participants who had poor social support were found to be around 60% (Table 3).

### 3.3. Types of Crime and Sentence Characteristics of Respondents

**Figure 1:**
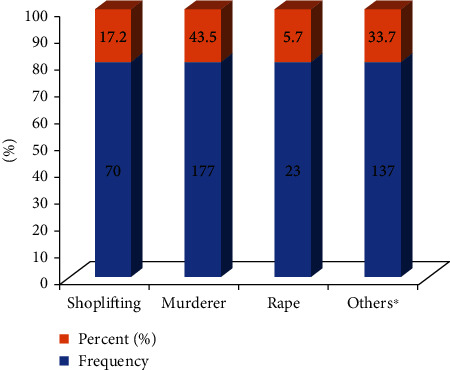
Types of crime among inmates in North Wollo Zone Correctional Institutions, Northeastern Ethiopia, 2021 (*n* = 407).

**Figure 2:**
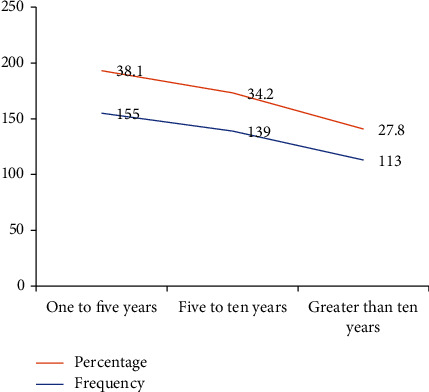
The length of sentences of inmates in North Wollo Zone Correctional Institutions, Northeastern Ethiopia, 2021 (*n* = 407).

Of the respondents, many of them were murderers (177 (43.5%)), and around one-quarter of them were sentenced to more than ten years (Figures 1 and 2). Around forty percent of study participants were sentenced to one- to five-year duration (Figure 2).

### 3.4. Factors Associated with Depression

In multiple logistic regression analysis, age from 38 to 47, having children, a prison sentence for 5-10 and more than 10 years, history of mental illness, having two or more stressful life events, and poor social support were significantly associated with depression. Prisoners aged 38-47 were four times more likely to experience depression than those aged 18-27 years (AOR = 4.29, 95%CI = 1.51, 12.20). Those who have children were almost three times more likely to experience depression as compared with those who have no children (AOR = 2.75, 95%CI = 1.40, 5.42). Inmates' sentence duration was another important factor. Prisoners sentenced for 5-10 years were six times more likely to develop depression (AOR = 6.26, 95%CI, 3.19, 12.30), and those who received a sentence of over 10 years were almost eight times (AOR = 7.71, 95%CI = 3.47, 17.17) more likely to have depression than inmates with sentence duration of 1-5 years. Prisoners with a history of mental illness were also five times (AOR = 5.22, 95%CI = 2.39, 11.36) more likely to have depression compared to inmates who have no history of mental illness. Having stressful life events was also associated with the odds of developing depression among prisoners. Prisoners with a history of two or more stressful life events were almost seven times (AOR = 6.61, 95%CI = 2.73, 15.96) more likely to develop depression than those who have no history of stressful life events. Furthermore, inmates with poor social support were eight times (AOR = 8.13, 95%CI = 3.43, 19.27) more likely to be depressed than those who have strong social support (Table 4).

## 4. Discussion

As to the researcher's knowledge, this is the first study conducted in the northeastern part of the country on the magnitude and determinants of depression among inmates. The prevalence of depression in our study was found to be 55.5%. This result is nearly similar to a study done in southern Ethiopian correctional institutions, 56.4% [8]. The possible reason for this being consistent with the south Ethiopian study might be due to the use of the same outcome measurement tool (Patient Health Questionnaire-9) and similar sociodemographic situations of the study participants. In addition, the two studies also took almost the same sample size and might have similar characteristics in the prison environment resulting in an approximate rate of depression prevalence among prisoners.

However, the current study finding is lower than studies done in Pakistan (85%) [35] and Egypt (82.5%) [36]. These variations might be due to differences in the depression assessment tool used, differences in socioeconomic and cultural situations, and different sample sizes. The Pakistan study revealed that they used 100 male participants and depression was assessed by a 21-item validated Urdu translation of the BECK depression inventory self-reporting questionnaire [35], while the Egyptian study used the screening tool SCL-90 and sample size (*n* = 80) [36]. Moreover, the cultural values and political contexts of these countries might have contributed to the difference in the prevalence rate of depression among prisoners, as political influence and lack of freedom are major reasons for depression [37]. On the hand, the present study result is higher than studies done in western countries and some other studies in Ethiopia: Nepal (35.3%) [38]; a meta-analysis report on global prevalence (36.9%) [2]; Jimma town, Ethiopia (41.9%) [39]; Nigeria (41%) [40]; Debre Birhan, Ethiopia (44%) [41]; Bahir Dar, Ethiopia (45.5%) [24]; Norway (46.1) [42]; and Ecuador (50.2%) [43]. A meta-analysis study in Ethiopia also reported a pooled prevalence of depression to be 44.5% [19]. The possible reasons for the current prevalence to be higher might be because of restriction of frequent contact with their families, the current devastating political situation of the country, lack of access to a library setting, crowded houses with poor ventilation and poor quality compared to others, banning from previous social activities, abundances of stressful life events in the prison, and restriction of movement and exercise [40, 44]. Concerning the factors, the present research revealed that there is a significant association between depression and poor social support. This finding is in line with a study done elsewhere [8]. The possible reason might be that inmates with depression are more prone to suffer in many domains of life and appear less likely to adapt to prison or life afterward. It also indicates that having a history of mental illness was significantly associated with the odds of developing depression among prisoners. This finding is supported by a previous study conducted in Addis Ababa city, Ethiopia [45]. This might be due to lack of social support and possible stigma from society, as feelings of exclusion or loneliness can bring on an episode in people who are prone to depressive disorders [31]. The present study further revealed that prisoners in the age groups of 38-47 years were more likely to develop depression than prisoners aged 18-27 years. This is consistent with a study finding from Jimma, Ethiopia [39]. The possible reason might be individuals in this age group are more likely to have an organized family, be more alcohol consumers, and use other substances before incarceration which might lead them to develop depression. It also revealed that the odds of having depression were higher among inmates who had two or more stressful life events compared to their counterparts. This is supported by a study report in which people who suffered from major life events such as divorce and loss of loved ones were found to be at risk of developing depression compared to those who have not experienced such events: Ethiopia [41] and Malaysia [16]. This study also showed that sentences for 5-10 years and more than 10 years were significantly associated with the odds of depression compared to sentences of less than 5 years. Consistent findings were reported from previous studies [24, 41]. This implies that prisoners with a long-term sentence are more likely to suffer from different symptoms of depression and despair, as compared to prisoners with a shorter sentence. This might indicate possible poor prison mental health services and could suggest the need for psychotherapists and psychiatrists in prison for proper management of depression among those prisoners sentenced to more than five years. It also revealed that having children had a significant association with depression. This might be because jailed parents may become worried about their son's or daughter's health, security, separation, well-being, and financial crisis. This in turn can make the parents feel guilty and lose interest which will further result in depression among prisoners [39].

### 4.1. Limitation

This study employed an interviewer-administered questionnaire to assess the prevalence of depression and its associated factors among prisoners. Therefore, participants may attempt to portray themselves in a more favorable way which is called social desirability bias. In addition, triangulating the study with qualitative methods could have been better to assess the effect of the political situation on the prevalence of depression among prisoners in a detailed way. However, this was not done due to time and budget constraints.

## 5. Conclusion

The prevalence of depression among prisoners was higher compared to many studies conducted across the globe. Inmates with age 38-47 years, having children, a sentence for 5-10 and above 10 years, a history of mental illness, having two or more stressful life events, and poor social support were significantly associated with the odds of developing depression among prisoners. Therefore, timely awareness creation and treatment programs about depression are required for those health professionals working at the institutions and for the inmates alongside the application of rehabilitation programs. In addition, routine screening of prisoners for depression and ensuring the availability of sufficient qualified personnel acting in full clinical independence and having sufficient expertise in psychology and psychiatry are recommended. These measures not only can help the prisoners in getting timely and appropriate care but also in preventing communities and families of inmates from potential harm during the reintegration process of prisoners after they are released from the prisons.

## Figures and Tables

**Table 1:** Sociodemographic characteristics of prisoners in North Wollo Zone Correctional Institutions, Northeastern Ethiopia, 2021 (*n* = 407).

Variables	Category	Frequency (*n*)	Percent (%)
Sex	Male	392	96.3
	Female	15	3.7

Age	18-27	167	41.0
	28-37	159	39.1
	38-47	45	11.1
	≥47	36	8.8

Marital status	Single	214	52.6
	Married	163	40.0
	Divorced	12	4.4
	Widowed	12	2.9

Religion	Orthodox	352	86.5
	Muslim	46	11.6
	Protestant	6	1.5
	Others^∗^	3	0.7

Ethnicity	Amhara	392	96.3
	Oromo	8	2.0
	Tigray	7	1.7

Occupation	Govt employed	51	12.5
	Farmer	126	31.0
	Merchant	47	11.5
	Unemployed	183	49.6

Residence	Rural	205	50.4
	Urban	202	49.6

Education	Cannot read and write	65	16.0
	Can read and write	342	84.0

Having children	No	194	47.7
	Yes	213	52.3

^∗^Catholic, Adventist, and Pagan.

**Table 2:** Clinical and prison-related factors among prisoners in North Wollo Zone Correctional Institutions, Northeastern Ethiopia, 2021 (*n* = 407).

Covariates	Category	Frequency	Percent (%)
Family history of mental illness	Yes	35	8.6
	No	372	91.4

Mental illness history	Yes	83	20.4
	No	324	79.6

Family history of alcohol use	Yes	90	22.1
	No	317	77.9

Medical history	Yes	33	8.1
	No	374	91.9

Number of crime records	One	335	82.3
	≥2	72	17.7

Sentences (years)	1-5	155	38.1
	5-10	139	34.2
	>10	113	27.8

Prison stay (years)	<1	205	50.4
	1 = 5	152	37.3
	>5	50	12.3

**Table 3:** Stressful life events and social support status among prisoners in North Wollo Zone Correctional Institutions, Northeastern Ethiopia, 2021 (*n* = 407).

Factors	Category	Frequency	Percent (%)
Stressful life event	None	67	16.5
	One	36	8.8
	Two or more	304	74.7

Social support	Poor	243	59.7
	Intermediate	98	24.1
	Strong	66	16.2

**Table 4:** Bivariate and multivariate logistic regression analysis of associated factors for depression among prisoners in North Wollo Zone Correctional Institutions, Northeastern Ethiopia, 2021 (*n* = 407).

Explanatory variables	Category	Depression		COR (95% CI)	AOR (95% CI)	*p* value
		No	Yes			
Age	18-27	114	53	1		
	28-37	46	113	5.284 (3.293, 8.479)	1.428 (0.715, 2.853)	0.31
	38-47	12	33	5.915 (2.832, 12.357)	4.288 (1.507, 12.198	0.006
	≥47	9	27	6.453 (2.837, 14.677)	2.083 (0.632, 6.862)	0.228

Educational status	Cannot read and write	31	34	1		
	Can read and write	150	192	1.167 (0.686, 1.986)	2.086 (0.942, 4.619)	0.070

Have children	No	121	73	1		
	Yes	60	153	4.227 (2.787, 6.411)	2.753 (1.400, 5.416)	0.003

Duration of sentences in years	1-5	110	45	1		
	5-10	51	88	4.218 (2.586, 6.879)	6.263 (3.187, 12.306)	0.001
	>10	20	93	11.367 (6.271, 20.602)	7.718 (3.469, 17.169)	0.001

Mental illness history	No	164	160	1		
	Yes	17	66	3.979 (2.237, 7.078)	5.213 (2.392, 11.362)	0.001

Stressful life events	None	51	16	1		
	One	18	18	3.187 (1.347, 7.544)	3.149 (0.997, 9.948)	0.051
	Two or more	112	192	5.464 (2.975, 10.037)	6.605 (2.733, 15.962)	0.001

Social support	Poor	60	183	11.329 (5.866, 21.880)	8.130 (3.431, 19.266)	0.001
	Intermediate	69	29	1.561 (0.750, 3.247)	1.793 (0.713, 4.510)	0.215
	Strong	52	14	1		

## Data Availability

The datasets used and/or analyzed during the current study are available from the principal investigator upon reasonable request.

## Abbreviations

AOR: Adjusted odds ratio
CI: Confidence interval
IRB: Institutional review board
PHQ-9: Patient Health Questionnaire-9
SSS: Social support scale.
